# Machine learning approaches for cancer prognosis and diagnosis via non-coding RNA: a comprehensive review

**DOI:** 10.1093/bib/bbag346

**Published:** 2026-09-07

**Authors:** Md Shafayet Hossain, Yi-Ping Phoebe Chen

**Affiliations:** Department of Computer Science and Information Technology, School of Computing, Engineering, and Mathematical Sciences, La Trobe University, Plenty Road, Bundoora, Melbourne, VIC 3083, Australia; Department of Computer Science and Information Technology, School of Computing, Engineering, and Mathematical Sciences, La Trobe University, Plenty Road, Bundoora, Melbourne, VIC 3083, Australia

**Keywords:** cancer, machine learning, non-coding RNA, miRNA, lncRNA, circRNA, piRNA

## Abstract

Non-coding RNAs (ncRNAs), once considered genomic dark matter, are now established as key regulators of gene expression with widespread roles in cellular homeostasis and disease. In cancer, ncRNA expression is frequently and systematically dysregulated, and many of these molecules circulate in stable, protected form within biofluids, offering a compelling basis for non-invasive or minimally invasive diagnostic strategies. However, their clinical translation remains substantially hindered to date due to biological complexity, technical noise, and high dimensionality inherent to ncRNA expression datasets. In this context, machine learning (ML) has emerged as a powerful analytical tool to address these challenges, enabling the identification of subtle, reproducible ncRNA signatures predictive of diverse malignancies. This review critically evaluates ML-driven frameworks for cancer diagnosis and prognosis across four ncRNA subclasses, namely miRNAs, lncRNAs, circRNAs, and piRNAs, while also acknowledging the biophysical and thermodynamic models that reinforce ncRNA bioinformatics. Despite substantial methodological progress in ML-based cancer diagnosis and prognosis, key challenges persist, including tumor biological heterogeneity, limited multicenter validation, and the lack of widely adopted standardized protocols for preprocessing, normalization, and reporting workflows. Furthermore, many current ML models lack interpretability in biological or clinical context, constraining their translational utility. By synthesizing recent advances and identifying unresolved barriers, this review charts a roadmap for developing a robust, clinically actionable ncRNA biomarker platform for cancer detection. With global cancer incidence projected to exceed 35 million annual cases by 2050, validated ncRNA-ML-driven frameworks hold potential to revolutionize early-stage detection and personalized therapeutic strategies, thereby reducing the escalating socio-economic burden of cancer worldwide.

## Introduction

**Figure 1:**
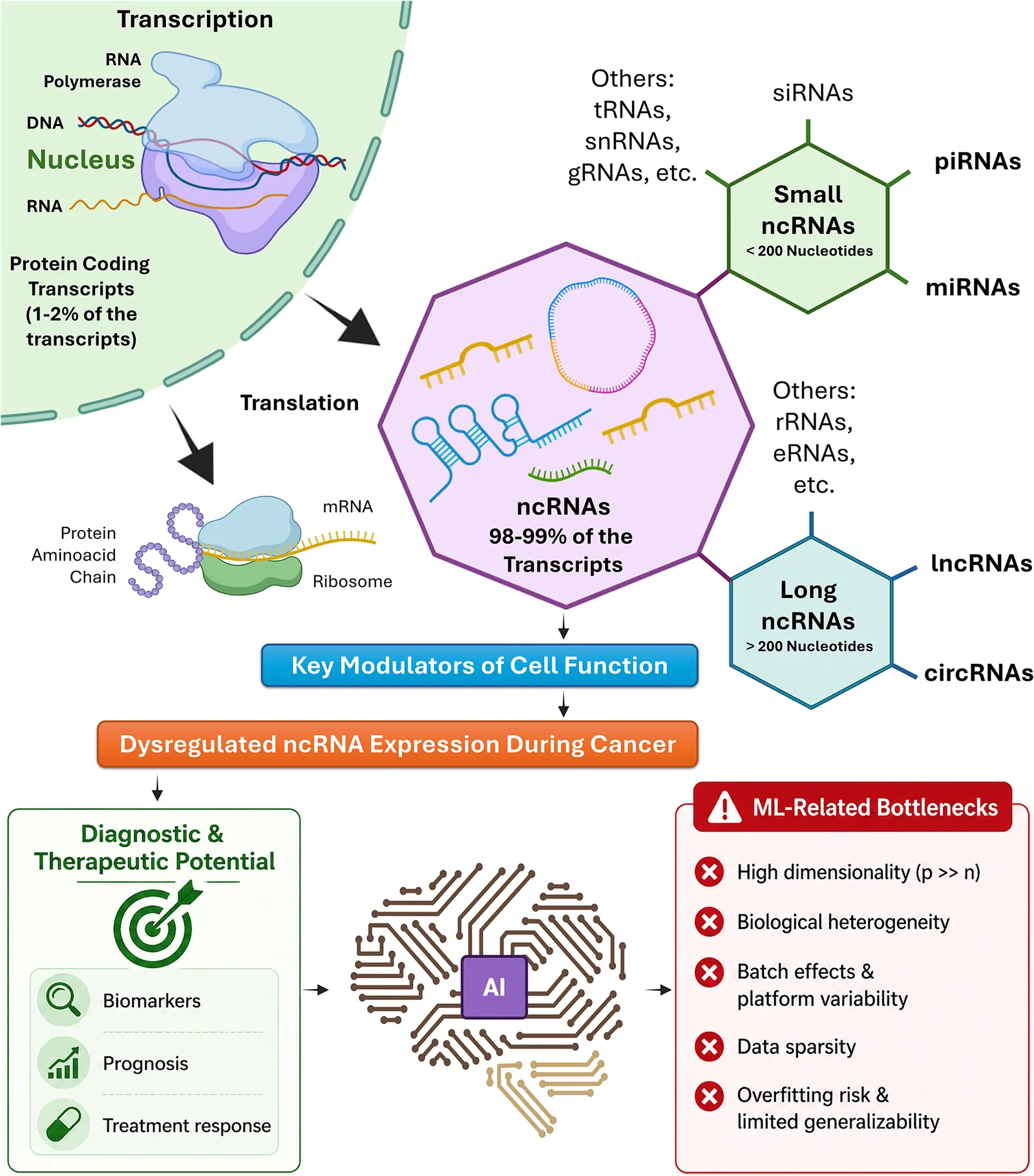
Transcriptomic landscape of ncRNAs along with ML-related bottlenecks. Abbreviations: eRNAs, enhancer RNAs; gRNAs, guide RNAs; rRNAs, ribosomal RNAs; siRNAs, small interfering RNAs; snRNA, small nuclear RNA; tRNAs, transfer RNAs.

Although roughly 75%–80% of the human genome undergoes biochemical activity, including transcription, a mere 1%–2% constitutes protein-coding sequences [1]. The RNA transcripts that are not translated into proteins are designated as non-coding RNAs (ncRNAs) and comprise approximately 98%–99% of the RNA transcripts [2, 3]. After their discovery, ncRNAs have been redefined from mere intermediates of translation to pivotal regulators of gene expression and genome architecture. High-throughput sequencing and allied technologies have progressively unveiled an expansive repertoire of ncRNA types varying in length and structure [4, 5]. This repertoire encompasses a heterogeneous array of transcripts, from long ncRNAs, which exceed 200 nucleotides, to small ncRNAs, defined as those shorter than 200 nucleotides (Fig. 1). Again, these two classes encompass micro RNAs (miRNAs), long non-coding RNAs (lncRNAs), circular RNAs (circRNAs), PIWI interacting RNAs (piRNAs), heterogeneous nuclear RNAs (hnRNAs), ribosomal RNAs (rRNAs), small nuclear RNAs (snRNAs), small nucleolar RNAs (snoRNAs), transfer RNAs (tRNAs), etc. [5] (Fig. 1).

ncRNAs constitute a valuable reservoir of next-generation biomarkers. Unlike the static nature of an individual’s DNA sequence, ncRNA expression exhibits high dynamism, with rapid modulation in response to diverse stimuli including cellular stressors. Consequently, an individual’s ncRNA signature can reflect the underlying molecular state, offering a mechanism-driven basis for biomarker discovery. Crucially, ncRNAs are not restricted to the intracellular environment; they are actively secreted into the circulatory system and other biofluids in stable forms [6]. These extracellular ncRNAs possess substantial translational application, given their accessibility via non-invasive sampling and the feasibility of cost-effective quantification using standard clinical laboratory methods [7].

Among the diverse ncRNA classes, miRNAs, lncRNAs, circRNAs, and piRNAs have been most intensively investigated for their roles in human pathology. These four classes have emerged as critical modulators of numerous cellular pathways, and their aberrant expression has been implicated in a spectrum of diseases, particularly cancers [8]. A central challenge, however, lies in precisely characterizing their diverse functions and mechanisms, which is a prerequisite for establishing their clinical utility as biomarkers for cancer prognosis and diagnosis.

Global cancer incidence is projected to surpass 35 million cases by 2050 [9], a clear indication of the critical need for early detection strategies to mitigate the considerable physical, psychosocial, and economic burdens placed on patients, healthcare infrastructures, and economies at both local and global scales. Despite this urgent demand, significant barriers exist. In the initial stages, primary tumors and metastases are often too small to produce systemic symptoms, rendering the disease clinically silent. Compounding this, the lack of screening assays that combine high specificity and sensitivity further hinders accurate cancer diagnosis.

Breakthroughs in functional genomics have substantially deepened the understanding of ncRNA biology and their contributions to the onset and progression of human cancers [2]. The first links between ncRNA dysregulation and cancer emerged in the early 2000s [10], and since then a rapidly growing body of evidence, driven by clinical diagnostic and concurrent advances in biosensor technologies and computational methodologies, has propelled intensive investigation of miRNAs, lncRNAs, circRNAs, and piRNAs as cancer biomarkers. Their stable presence in biofluids such as blood, saliva, and urine further positions them as compelling candidates for cancer diagnosis and prognosis.

Precision oncology demands early and accurate cancer detection, yet conventional bulk transcriptomic analyses often conceal critical molecular heterogeneity and fail to translate into improved patient outcomes. The high-dimensional expression profiles and pervasive data sparsity of ncRNA datasets present considerable analytical difficulties that conventional statistical methods struggle to address. In this context, machine learning (ML) offer the capacity to extract robust, linear, and nonlinear patterns from such datasets, and integrate complementary modalities such as single-cell and spatial transcriptomics. By harnessing these computational strengths, researchers can uncover subtle malignancy signatures and accelerate the translation of ncRNA biomarkers into clinically actionable assays.

The bioinformatic analysis of ncRNAs is, however, grounded in biophysical models that have been developed over decades and remain fundamentally important, not as methods that ML has replaced, but as indispensable frameworks that ML methods routinely depend upon and complement. RNA secondary structure prediction via free energy minimization, implemented in landmark tools such as Mfold [11] and the ViennaRNA Package [12] and parameterized using the Turner nearest-neighbor thermodynamic energy model [13], constitutes the computational basis for understanding miRNA hairpin processing, lncRNA structural domain architecture, and RNA duplex stability. miRNA target identification, a prerequisite for interpreting cancer-associated miRNA signatures, relies on thermodynamics-based algorithms such as TargetScan [14] and miRanda [15], which evaluate seed complementarity and duplex free energy. Similarly, the discovery of circRNAs in cancer depends on back-splice junction detection algorithms such as find_circ [16] and CIRI [17], while piRNA cluster annotation is grounded in probabilistic biophysical frameworks such as proTRAC [18]. Together, these models define the biological and structural feature space, encompassing hairpin stability, target accessibility, back-splice geometry, and ping-pong signatures. It is equally important to acknowledge that prior to the advent of ML-based classification, ncRNA expression data were analyzed almost exclusively through statistical and biophysical modelling approaches that remain foundational reference points in the field. Differential expression analysis coupled with Cox proportional hazards regression and Kaplan–Meier survival modelling first established the prognostic relevance of miRNA signatures across multiple solid tumors [19]. Hazard ratio-based survival frameworks similarly confirmed lncRNAs such as HOTAIR and MALAT1 as independent prognostic factors across diverse cancer types [20], while Mitchell *et al.* [21] demonstrated that circulating miRNAs are detectable and quantifiable in serum as stable, non-invasive cancer biomarkers using logistic regression and ROC analysis. These non-ML studies not only laid the clinical and biological groundwork for biomarker discovery but also defined the performance benchmarks against which ML models must be measured. Collectively, these biophysical frameworks constitute the benchmark of ncRNA bioinformatics, and therefore their continued relevance in ncRNA bioinformatics must be understood as foundational rather than peripheral [2].

The objective of this review is to assess the promise and limitations of ML-driven strategies for cancer diagnosis and/or prognosis using ncRNAs, specifically miRNAs, lncRNAs, circRNAs, and piRNAs as clinically actionable biomarkers. Accordingly, only studies published in peer-reviewed journals are considered, with eligibility limited to ML-based investigations focused on cancer diagnosis and/or prognosis while excluding ‘Disease-association’ studies. To the best of our knowledge, this is the first comprehensive survey in which four distinct classes of ncRNA-based cancer detection studies employing ML algorithms are critically evaluated and analyzed. Both methodological and conceptual constraints of existing studies are examined, and directions for future investigation are proposed. In doing so, this review describes the current landscape of ML-based cancer detection using ncRNAs, examines persistent challenges, and charts a roadmap for future innovations in ncRNA-driven cancer prognosis and diagnosis.

## Mechanisms of action and functions of ncRNAs

By structural characteristics, modes of action, biological functions, and length, ncRNA transcripts are classified into two main classes: small and long ncRNAs.

Small ncRNAs comprise miRNAs along with PIWI interacting RNAs (piRNAs), small nuclear RNAs (snRNAs) that mediate splicing, small nucleolar RNAs (snoRNAs) involved in rRNA modification, tRNA derived small RNAs (tsRNAs), YRNAs (RNY), and YRNA derived small RNAs (YsRNAs; s RNY), etc. [22, 23]. YRNAs are transcribed by RNA polymerase III and involved in DNA replication, RNA quality control, and stress responses. Under apoptotic conditions, they are cleaved independently of the Dicer/miRNA pathway into YsRNAs [24]. Both YRNAs and YsRNAs are detectable and circulate stably in plasma and serum. In several clinical studies, dysregulated YRNA expression has been reported across multiple cancer types, including breast, bladder, prostate, lung, and brain tumors [24].

Long ncRNAs include both linear lncRNAs and circular RNAs (circRNAs). Moreover, a subset of ‘coding lncRNAs’, whose sequences encode functional micro peptides, has been identified and shown to regulate vascular and cardiac hypertrophic responses [25, 26].

In Table 1, miRNA, lncRNA, circRNA, and piRNA are compared with respect to their mechanisms of action and functions [27–31].

**Table 1:** Comparative overview among miRNA, lncRNA, circRNA, and piRNA.

Feature	miRNA	lncRNA	circRNA	piRNA
Length	~18–24 nucleotides	>200 nucleotides (often 1–10 kb)	Typically, ~200–800 nucleotides	~24–31 nucleotides
Annotation (human)	~2.6k (miRBase v22.1)	~94.8k (NONCODE v7.0)	~32.9k (circRNADb)	~32.8k in piRBase
Genomic origin	Discrete hairpin loci (inter-/intragenic)	Intergenic, antisense or intronic transcripts	Back-splicing of pre-mRNA	Long genomic clusters
Stability	Moderate	Generally low	Very high	Highly stable in germ cells
Cellular localization	Cytoplasm, nucleus, extracellular vesicles (EVs)	Nucleus, cytoplasm, some EVs	Mostly cytoplasmic; some nuclear retention	Cytoplasm and nucleus
Primary mechanisms of action	Binds mRNAs 3′UTR	Scaffolding of proteins	Sponges’ miRNAs	Guides PIWI proteins
Role in cell–cell communication	Packaged in EVs; act as intercellular/paracrine signals	Some lncRNAs are secreted in EVs (functions less defined)	Abundant in exosomes	Primarily intracellular (germline defense); EV role not established
Tissue specificity	Many ubiquitous; some show tissue- or stage-specific patterns	Highly tissue- and context-specific	Variable: some ubiquitous, others cell-type-specific	Strongly germline-enriched; few somatic piRNAs known
Typical abundance	Moderate-high (hundreds to thousands of copies per cell)	Low (often <10 copies per cell for many lncRNAs)	Can accumulate to high levels due to stability	Very high in germ cells; low in most somatic cells
Clinical relevance	Widely explored as cancer biomarkers in liquid biopsies	Widely explored as disease biomarkers in tissue	Emerging cancer biomarkers in biofluids	Investigated as cancer epigenetics; less explored
ML application in cancer	Widely used and well-established	Increasingly used	Growing	Rarely used in current ML pipelines; has potential


## ncRNA dysregulation in cancers and ML

ncRNAs execute regulatory functions across virtually all cellular pathways, and their abnormal expression is now indisputably implicated in the pathogenesis of diverse cancers. In 2002, genetic analyses of chronic lymphocytic leukemia (CLL) uncovered that deletion of the chromosome 13q14 locus (the genomic region encoding pivotal miRNAs) constituted, the solitary recurrent lesion in leukemic clones in many cases [10]. This pivotal observation affirmed ncRNAs as bona fide oncogenic mediators and catalyzed intensive investigation into their prospective roles as both diagnostic indicators and therapeutic targets for cancers.

Several intrinsic properties establish ncRNAs as highly promising biomarker. First, pathological specimens routinely manifest reproducible, disease-specific dysregulations in ncRNA abundance and activity, thereby providing a precise molecular fingerprint of underlying pathology [32]. Second, a comprehensive arsenal of analytical modalities (from quantitative PCR and hybridization platforms to high-throughput RNA sequencing) enables reliable quantification of small and long ncRNAs in heterogeneous samples. Third, many ncRNAs are secreted into extracellular vesicles or move freely in biofluids (including plasma, urine, saliva, cerebrospinal fluid, and breast milk) [33], thus facilitating minimally invasive liquid biopsy.

Despite the numerous annual publications that document dysregulated ncRNA expression in oncology, only a select few candidates have entered clinical evaluation [as tracked by the NIH Early Detection Research Network (EDRN) and ClinicalTrials.gov]. This translational chasm arises from multifaceted obstacles. Beyond the constant challenges of assay reproducibility and sample heterogeneity, a critical impediment resides in current data-analytic paradigms. Conventional methods frequently collapse rich ncRNA datasets into rudimentary fold-change thresholds or univariate correlations with clinical endpoints, thereby neglecting the nuanced interplay among patient demographics, disease phenotypes, and molecular networks.

Addressing this complexity mandates integrative models that synthesize ncRNA metrics with data such as patient age, comorbidities, therapeutic exposures, and even biological sex, given its documented influence on disease trajectory. Embedding high-dimensional ncRNA signatures within electronic health records, in conjunction with established biomarkers, promises to reveal novel prognostic and predictive axes and to expedite clinical translation [34]. In this light, ML has become a compelling choice to remedy these shortcomings. By training sophisticated algorithms on expansive, multi-dimensional datasets, ML can detect intricate nonlinear relationships and higher-order feature interactions that elude conventional statistical techniques. Such approaches adeptly integrate molecular variables with sociodemographic and phenotypic data, deconvoluting the elaborate regulatory schemas governed by ncRNA networks. Consequently, ML-driven pipelines are swiftly becoming indispensable for prioritizing candidate ncRNAs and constructing robust predictive signatures.

**Figure 2:**
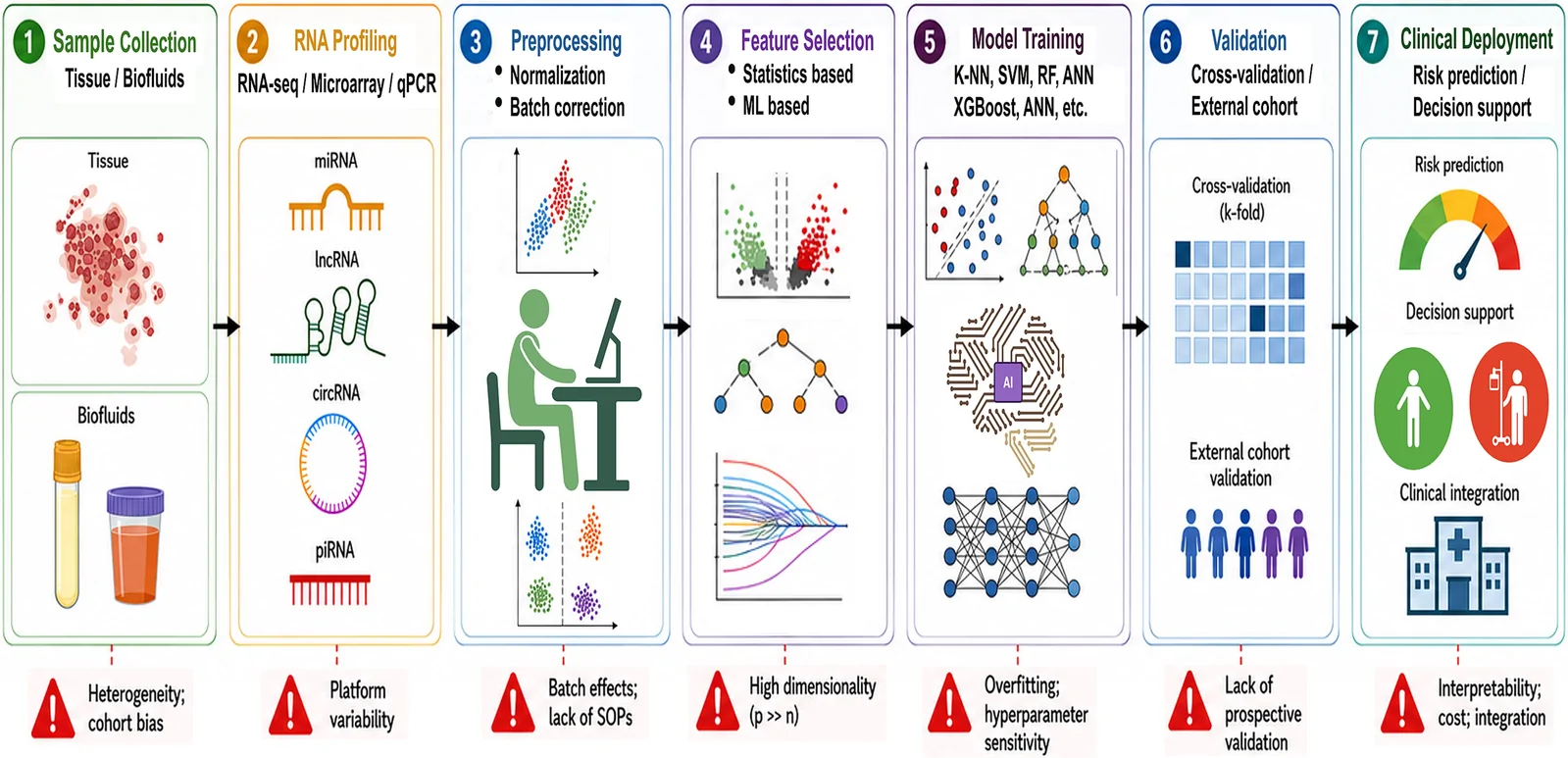
An end-to-end ncRNA based cancer diagnosis pipeline using ML highlighting the key methodological bottlenecks.

An end-to-end ncRNA-based cancer diagnosis pipeline using ML is illustrated in Fig. 2, which is self-explanatory. However, critical bottlenecks arise at multiple stages of this pipeline, including cohort bias and biological heterogeneity during data acquisition, platform-dependent variability during RNA profiling, which compromises measurement consistency and reproducibility; and batch effects resulting from the absence of standardized operating procedures (SOPs). Furthermore, the high-dimensional, low-sample-size nature of ncRNA datasets (p >> n) is likely to complicate feature selection and increases the risk of overfitting, particularly in small cohorts or under suboptimal hyperparameter tuning. Finally, inadequate external and prospective validation remains a major barrier to clinical reliability.

## Supervised ML methods in ncRNAs-based cancer detection

Several data acquisition methodologies such as quantitative PCR, microarrays, and next-generation sequencing have produced vast transcriptomic datasets, thereby necessitating comprehensive feature extraction from large-scale datasets. In recent years, the application of ML algorithms to high-dimensional ncRNA profiles has expanded rapidly. Table 2 outlines most prevalently used ten supervised ML algorithms, i.e. K-Nearest Neighbors (KNN), Naïve Bayes, Decision Trees, Random Forest (RF), Extreme Gradient Boosting (XGBoost), Artificial Neural Network (ANN), Support Vector Machine (SVM), Least Absolute Shrinkage and Selection Operator (LASSO), Ridge Regression, and Elastic Net highlighting their core principle, strengths, limitations, performance, and computational efficiency.

**Table 2:** Most prevalent supervised machine learning methods in cancer prognosis and diagnosis utilizing ncRNAs; based on current publications.

Method	Core principle	Advantages	Limitations	Performance	Computational efficiency
KNN	Distance-based instance learning	Simple; no training phase	Requires tuning; poor scalability	High with well-separated classes	No explicit training required; slow prediction in high-dimensional datasets
Naïve Bayes	Probabilistic (independence assumption)	Fast, effective on small datasets	Assumption of conditional feature independence	High under feature independence; declines with correlated features	Very fast as class probabilities derived directly without iterative optimization.
Decision Trees	Recursive feature partitioning	Interpretable; handles nonlinearity	Overfitting and underfitting risk; instability	Excellent training performance; susceptible to overfitting on unseen data	Generally fast; training/inference time increases with tree size
Random Forest	Ensemble of bootstrapped trees	Robust; handles high-dimensional data	Reduced interpretability	Robust performance with proper hyperparameter tuning	Computational cost increases with number of trees and depth
XGBoost	Regularized gradient boosting	State-of-the-art performance	Requires tuning; less interpretable	Very high with optimal hyperparameter tuning	Exceptionally swift and resource-light.
ANN	Multi-layer nonlinear mapping	Captures complex patterns	Data-intensive; overfitting and underfitting risk	High with ample data and well-tuned hyperparameters	Slow training for complex models; fast inference
SVM	Margin maximization with kernels	Effective in high dimensions	Critical kernel selection	High with appropriate kernel and hyperparameter tuning	Slow training with non-linear kernels; fast inference
LASSO	L1-regularized linear model	Sparse feature selection	Performs poorly with correlated features	High with optimal hyperparameter tuning	Moderate to high cost during training; fast inference
Ridge regression	L2-regularized linear model	Handles multicollinearity	No feature selection	High with finely tuned hyperparameter	Faster training than LASSO; fast inference
Elastic Net	Combined L1 + L2 regularization	Balances sparsity & multicollinearity	Requires extensive parameter tuning	High with proper hyperparameter tuning	Higher computational cost than LASSO and Ridge

Since each model architecture presents unique advantages and constraints, selecting an appropriate algorithm demands a careful balancing of competing considerations. This choice hinges not only on dataset characteristics but also on research objectives, available computational resources, and the necessity for model interpretability. Comprehensive benchmarking of multiple ML frameworks is essential to identify the most suitable approach for a given investigative context.

## Application of ncRNA-ML-driven cancer diagnosis and prognosis

A growing body of research indicates that the application of supervised ML methodologies holds considerable promise for the discovery of ncRNA-based cancer prognosis and diagnosis, the construction of predictive classifiers to aid clinical decision-making, and the deeper investigation of the molecular mechanisms underlying disease pathogenesis.

### miRNA, ML, and cancer

In this subsection, the most recent and impactful studies that used miRNA and ML approach in cancer diagnosis are discussed (Table 3).

**Table 3:** Summary of cancer diagnosis studies using miRNA and ML.

Study design/reference	Cancer type	Sample matrix	Sample size/dataset availability/code availability	Preprocessing and feature selection	Machine learning model	Performance metrics
Developed and validated 6-cf/exo-miRNA-based panel for early-onset colorectal cancer detection across an international multicenter, prospective cohort/[35]	Colorectal cancer	Plasma and tissue	621 samples from 4 independent clinical cohorts & GEO database/ Partial/No	Statistical significance filtering and LASSO regression	XGBoost	AUC (0.96), sensitivity (0.92), specificity (0.88)
Proposed a 10-miRNA signature Destinex assay for early GC detection through a multicenter, case-controlled test subjects/[36]	Gastric cancer	Serum	809 samples from 4 clinical cohorts/No/No	DE analysis, overlap filtering, and LR for feature selection	XGBoost	AUC (0.95), sensitivity (0.89), specificity (0.89)
Developed a 6-miRNA & nodule size based framework showing strong potential as a non-invasive alternative to LDCT for lung cancer screening/[37]	Lung cancer	Serum	205 samples (82 cancer, 123 controls)/No/No	RF for feature selection	KNN, NNET, SVM, Naïve Bayes	AUC (0.99), sensitivity (0.98), specificity (0.98)
Proposed 100-miRNA and CA19-9-based model for early-stage pancreatic cancer detection validated in asymptomatic cases/[38]	Pancreatic cancer	Serum	425 samples (213 cancer, 212 controls)/No/No	Normalization using ComBat, Criterion based feature selection	AutoML platform DataRobot	AUC (0.99), sensitivity (0.98), specificity (0.98)
Proposed 3-miRNA-based generalizable, non-invasive screening for breast cancer from both healthy and 11 other cancer types/[39]	Breast cancer	Serum	11,349 samples from GEO database/ Yes/No	Normalization, feature selection using IV, RF, MRMR, & LASSO	SVM, RF, LR, XGBoost, AdaBoost, GB	AUC (0.99), sensitivity (0.96), specificity (0.98)
Developed cancer (16 types) vs non-cancer detection model using RNA-modification-targeted 79-miRNA with optimized ML workflow/[40]	Cancer versus non-cancer	Serum	43 047 samples (20 021 cancer, 23 026 controls) from GEO database/Yes/No	Normalization, batch correction (ComBat), DE analysis, feature reduction via LASSO & RFE.	SVM, KNN SGBT, XGBoost, RF, NNET, avNNET, MARS, Naïve Bayes	AUC (.99), sensitivity (0.99), specificity (0.93)
Proposed cell-free immune-related 39-miRNA-based classifier for robust early detection of cancers (13 types) with strong performance in early stages/[41]	Cancer versus non-cancer	Serum	15 832 samples from GEO database +684 samples for external validation/Yes/No	Immune-related miRNA curation, Differential expression filtering, LASSO for feature selection	XGBoost, LR, SVM, RF, LASSO	AUC (0.98), sensitivity (0.93), specificity (0.95)
Developed a 3-miRNA signature model that accurately distinguishes pancreatic cancer from both healthy controls and pancreatitis cases/[42]	Pancreatic cancer	Serum & tissue	3692 samples (3265 PC,427 control) + own lab (13 PC,13 HT)/Partial/No	Batch correction (sva), differential expression analysis, RRA feature ranking	RF, CART, SVM, LR	Accuracy (0.94), sensitivity (0.95), specificity (0.80)
Developed 60-miRNA-based self-organizing deep neuro-fuzzy model for the classification of 5 kidney cancer subtypes/ [43]	Kidney cancer subtypes	Tissue	1071 samples across 5 kidney cancer subtypes from TCGA database/Yes/No	Data cleaning, correlation-based feature selection using AMGM	Self-organizing deep neuro-fuzzy system	Accuracy (0.93), sensitivity (0.92), specificity (0.98)
Demonstrated 15-miRNA-based diagnostic model for symptomatic lung cancer patients/[44]	Lung cancer	Whole blood	3046 individuals (606 lung cancer, 2440 mixed control/disease)/No/No	Normalization, ANOVA filtering	Gradient boosted trees	Accuracy (0.91), sensitivity (0.83), specificity (0.94)

Mannucci *et al.* [35] conducted an international, multicenter, prospective cohort investigation enrolling 542 participants (621 samples) from the USA, Italy, Spain, and Japan to develop and validate a blood-based liquid biopsy test for early-onset colorectal cancer detection study diagnosed before age 50. The study adhered to the EDRN phased framework, progressing through a small RNA sequencing discovery cohort (*n* = 118), an ML training cohort (*n* = 192), and an independent external testing cohort (*n* = 191), with a post-surgical monitoring sub cohort (*n* = 41). From 442 cell-free and 205 exosomal miRNA candidates, LASSO regression and multi-step biological filtering identified a 6-miRNA panel comprising two cell-free miRNAs (hsa-miR-625-3p and hsa-miR-30d-5p) and four exosomal miRNAs (hsa-miR-32-5p, hsa-miR-486-3p, hsa-miR-550a-3-5p, and hsa-miR-625-3p). An XGBoost model trained on RT-qPCR measurements of this panel achieved Area Under the Curves (AUCs) of 97.5% in training and 95.6% in independent testing. In the testing cohort, overall accuracy, specificity, positive predictive value, and negative predictive value were 89.5%, 87.5%, 87.9%, and 91.3%, respectively, with stage-specific sensitivity of 94.1% (stage I), 100.0% (stage II), and 96.8% (stage III). Importantly, biomarker levels declined significantly following curative surgery, supporting tumor-origin specificity. One limitation of this study is the absence of cost-effectiveness data and lack of head-to-head comparison with stool-based RNA tests or colonoscopy, collectively tempering immediate clinical translation.

Sui *et al*. [36] presented a rigorously designed, four-phase multicenter case–control study aimed at developing a blood-based, exosomal miRNA signature for early gastric cancer (GC) detection. Using genome-wide small RNA sequencing of 47 tissue, 32 exosomal, and 43 cell-free RNA samples from GC patients and controls, the authors identified 10 exosomal and 8 cell-free miRNAs enriched in GC. An XGBoost classifier was then applied to the training cohort (*n* = 263) to develop a 17-miRNA combination signature (eight cell-free and nine exosomal) that achieved an AUC of 0.96 and was subsequently reduced to a clinically streamlined 10-miRNA panel called Destinex (five cell-free and five exosomal miRNAs). Validated in an independent cohort of 217 samples across four major Asian cancer centers, Destinex achieved an AUC of 0.95, sensitivity of 0.89, and specificity of 0.89, with superior early-stage (pT1) detection performance (AUC = 0.97). Postsurgical decreases in miRNA expression confirmed tumor-specific origin, and SHAP values enhanced interpretability by quantifying individual miRNA contributions. The study’s principal strength is its multi-institutional multinational design, four-phase biomarker development workflow, and demonstration of early-stage diagnostic utility. However, the cohort was restricted exclusively to Asian participants, limiting generalizability to other populations. Patients with high-risk precursor lesions such as chronic atrophic gastritis and intestinal metaplasia were not enrolled, and the sample size for comparative evaluation against other gastrointestinal cancers was limited. Prospective validation in ethnically diverse, population-based screening cohorts remains essential before routine clinical deployment.

The authors of [37] presented a well-designed case-controlled investigation using serum miRNA signatures to classify lung cancer. The expression profile data of six miRNAs (miR-196a-5p, miR-1268, miR-130b-5p, miR-1290, miR-106b-5p, and miR-1246) coupled with radiological nodule dimensions were utilized to construct two separate predictive ML-based frameworks for the early detection of lung cancer. The study involved analysis of serum samples from 205 patients (82 patients with lung cancer and 123 individuals as controls) enrolled between 2020 and 2023 from two Singaporean tertiary hospitals. Initially, the authors shortlisted 25 candidate miRNAs based on the literature review, narrowed them down to 6 using expression analysis and Random Forest feature ranking, and quantified them using qPCR. Two different frameworks (one consisting of miRNAs alone and the other consisting of miRNAs with nodule size) were developed using several ML algorithms (Table 3). Despite the impressive KNN-derived AUC of 0.989 (sensitivity 92.1%, specificity 97.5%) for the combined model, the reliance on a small cohort (*n* = 205) from two Singaporean centers constrains external validity and raises the possibility of overfitting, particularly given the absence of an independent validation set. Although the comparative evaluation of several ML models (KNN, NNET, SVM, and Naïve Bayes algorithms) and the rigorous five-fold cross-validation ensure methodological robustness, the exclusive use of R-derived Ct values without orthogonal validation (e.g. digital PCR) and the initial shortlisting of miRNAs through literature review may accidentally exclude novel, low-abundance miRNA markers. Hence, the proposed model’s generalizability across ethnically diverse or low-dose computed tomography (LDCT) ineligible populations could be restricted.

In Kawai *et al.* [38], the development of a diagnostic model for early-stage pancreatic cancer by integrating serum miRNA profiles with CA19–9 levels was reported. This study analyzed 425 serum samples (213 from pancreatic cancer patients and 212 from healthy controls) sequenced for 2588 miRNAs, from which 100 robust, highly expressed miRNAs were selected and normalized. Using AutoML, the authors developed and validated several ML models, including ensemble methods, with performance evaluated on an independent validation cohort. The combined model (miRNA and CA19-9) achieved an AUC of 0.99, 67% sensitivity for stage 0–I detection, >90% for advanced stages, and 98% specificity. The modest sample size (*n* = 425) collected from 14 different hospitals, with independent validation and orthogonal NGS platforms boosted internal credibility. However, the reliance on retrospective cohorts and algorithmic ‘black box’ ensembling could raise concerns about overfitting and model interpretability. Moreover, the modest 67% sensitivity for stage 0–I disease is a clear indication of the need for further optimization and prospective validation in ethnically diverse, population-based screenings to confirm clinical utility and cost-effectiveness.

The study [39] aimed to establish an accurate, non-invasive breast cancer screening model using serum miRNAs to distinguish breast cancer from healthy controls and 11 other cancer types in a large, heterogeneous population. The dataset contained a total of 11 349 serum miRNA samples (GSE211692 and GSE730002) with internal and external validations. By applying three different feature selection techniques, namely MRMR, LASSO, and Random Forest, the authors reduced 2550 miRNAs to the top 3 candidate miRNAs: miR-1307-3p, miR-5100, and miR-4745-5p. Both models, SM4BC3miR (AdaBoost-based) with raw miRNA values and SSM4BC (Random Forest-based) with expression ratios, were designed, and both models provided high AUCs across test, internal, and external sets. The study’s strongest contribution is its proof of efficient cancer discrimination with only three miRNAs validated in one of the largest real-life cohorts assembled up until now. However, the slight disparity between training and test metrics could raise the concern of overfitting, particularly as the reliance on retrospective GEO datasets cannot fully account for center-specific preanalytical variability, demographic heterogeneity, or unmeasured confounders. Moreover, although ratio-based normalization mitigates batch effects, the exclusive focus on three miRNAs might prevent exploration of potentially informative low-abundance miRNA transcripts. In addition to this, the absence of prospective, ethnically diverse clinical trials, absent cost-effectiveness analyses, and mechanistic validation further limits translation to routine screening.

The study reported in [40] developed a non-invasive cancer diagnostic model using serum miRNAs with mutation-targeted RNA modification. Using 43 047 serum samples [consisting of 20 021 cancerous (16 types) patients and 23 026 non-cancer controls] obtained from the publicly available GEO database, the authors investigated 504 RMvar-related miRNAs to classify cancer from non-cancer using nine types of ML algorithms. From differential expression (DE) and enrichment analysis, 298 candidate miRNAs were selected for initial analysis and reduced further to 79 miRNAs using Recursive Feature Elimination and LASSO techniques. Among the nine different ML models, the best performance was reported using SGBT and SVM and these two models were merged into an ensemble classifier using caretEnsemble with an AUC of 0.998, a sensitivity of 99.3%, and a specificity of 93.1% against external validation sets. The most significant contribution of the study is the ensemble model that correctly classified early-stage cancers using biomarkers found in blood. However, the large miRNA panel size, absence of prospective, multicenter clinical trials, and orthogonal validation (e.g. digital PCR or mass spectrometry for modified nucleotides) constrains immediate translational promise.

In [41], the authors aimed to establish cf-IRmiRNAs as a reliable non-invasive biomarker for early cancer detection across multiple cancer types. Using 15 832 plasma-derived samples from both cancer patients (13 types) and non-malignant controls, the authors employed a robust preprocessing pipeline followed by LASSO regression to narrow down 1256 candidate miRNAs to 39 key cf-IRmiRNAs. The final diagnostic model was constructed using the XGBoost algorithm, which outperformed other four ML classifiers (LR, LASSO regression, SVM, and RF), achieving exceptional diagnostic accuracy with AUC values reaching 0.984 in validation and 0.983 in test set. The classifier demonstrated impressive performance in early-stage cancer detection and benign-malignant differentiation, particularly for lung, gastric, and prostate cancers. The study’s strongest contribution lies in its scale and the diagnostic robustness of its cf-IRmiRNA-based classifier. Also, the rigorous 7:2:1 split, five-fold cross-validation and independent external testing highlight internal consistency, while pathway enrichment strengthens biological plausibility. However, the reliance on flag-based abundance thresholds and the retention of 39 miRNAs, though more manageable than genome-wide panels, still poses substantial challenges for clinical translation and practice.

The authors of [42] proposed a minimally invasive diagnostic approach for early-stage pancreatic cancer using a 3-miRNA serum signature (hsa-miR-1246, hsa-miR-205-5p, and hsa-miR-191-5p). The investigators presented a multi-dataset integration of 11 different miRNA expression cohorts from the publicly available GEO database, and subsequently trained and validated four machine-learning classifiers (RF, SVM, CART, LR) on real-world serum specimens, yielding testing accuracy up to 94.4% and real-world validation performance of 83.5% (91.5% accuracy in pancreatitis versus cancer discrimination). The inclusion of functional enrichment and target prediction convincingly implicated hsa-miR-205-5p in TGF-beta and Wnt pathway dysregulation and thereby offered both diagnostic and therapeutic insight. But the small sample size of the clinical validation set (*n* = 55), and potential confounding from heterogeneous data platforms and batch effects are potential shortcomings of this study for real-life clinical translation. Also, these shortcomings warrant the necessity for larger, ethnically diverse, prospective trials to ascertain robustness, generalizability, and mechanistic fidelity of their proposed model.

In [43], using 60 candidate miRNA expression profiles, the authors developed a fairly accurate and interpretable ML-based system that classified kidney cancer subtypes. Using The Cancer Genome Atlas (TCGA) and TARGET database data of 1881 miRNAs in five kidney cancer subtypes, the authors used a three-step pipeline that included preprocessing, selection of features using the arithmetic mean-geometric mean (AMGM) dispersion measure, and a deep neuro-fuzzy self-organizing classifier. Their proposed model achieved 93.2% accuracy and outperformed logistic regression, decision trees, and LSTM models with 92.4% sensitivity, 98.1% specificity, and MCC of 89%. Their use of Gaussian membership functions and a rule-learning autoencoder enhanced noise resilience and interpretability, while the comparative benchmarking against LSTM, decision trees, and logistic regression highlighted performance gains. However, the absence of external, multicenter clinical validation (especially for rarer subtypes such as Wilms and rhabdoid tumors), the retrospective design, and the potential for overfitting given the complex self-organizing architecture might hinder translational possibilities. Despite claims of interpretability, the optimized fuzzy rules and deep encoding layers may still obscure mechanistic insight, necessitating further wet-lab validation and simplification of rule structures before clinical deployment.

The study [44] took a closer look at whether blood-based miRNA patterns can help identify lung cancer in patients already showing symptoms. It comprised retrospective, multicenter analysis of 3046 symptomatic patients (606 with lung cancer, 593 with non-tumor pulmonary disease, 883 with unrelated conditions, and 964 healthy controls) leveraging quantile-normalized microarray profiling of 1183 miRNAs and a LightGBM-driven feature selection technique to construct an ML-based classifier. The authors reported 91.4% accuracy, 82.8% sensitivity, and 93.5% specificity in screening lung cancer from control. However, the reliance on heterogeneous PAXgene processing protocols across five institutions and nearly decade-long sample collection (2009–2018) might introduce preanalytical variability, while retrospective age-, sex- and smoking-matched cohorts cannot fully mitigate selection bias, and the absence of independent, prospective validation (particularly in external high-risk screening populations) limits generalizability. Moreover, although permutation tests and LIMMA-based batch corrections bolster internal validity, the ‘black-box’ nature of gradient boosting impedes biological interpretability of the selected miRNAs, warranting the need for orthogonal validation using digital PCR or sequencing, and mechanistic studies to clarify miRNA origins in tumor biology to confirm clinical utility before adoption as a complement or alternative to low-dose CT screening.

### lncRNA, ML, and cancer

In this subsection, the most recent and impactful studies that used lncRNA and ML in the prognosis of cancers are discussed (Table 4).

**Table 4:** Summary of cancer prognosis studies using lncRNA and ML.

Study design/reference	Cancer type	Sample matrix	Sample size/dataset availability/code availability	Preprocessing and feature selection	Machine learning model	Performance metrics
Proposed 5-lncRNA-based prognostic model for GC patients validated across multiple clinical parameters and enrichment pathways/[45]	Gastric cancer	Tumor tissue	371 samples from TCGA database/Yes/No	Co-expression analysis, univariate and LASSO Cox regression	LASSO regression	AUC (1-yr - 0.72), (3-yr - 0.65), (5-yr - 0.69)
Constructed 16-lncRNA-based prognostic model for GC with immune microenvironment characterization/[46]	Gastric cancer	Tumor tissue	835 samples from TCGA and GEO databases/ Yes/No	Batch correction, consensus clustering LASSO-univariate and multivariate Cox	Multivariate Cox regression; LASSO	AUC (1-yr - 0.67), (3-yr - 0.68), (5-yr - 0.66)
Developed 27-lncRNA-based prognostic model for LUAD highlighting role of DRLs in immune landscape/[47]	Lung adenocarcinoma	Tumor tissue	916 samples from TCGA and GEO databases/Yes / No	Normalization, batch correction using ComBat, univariate cox regression	Ridge, LASSO, Stepwise Cox, CoxBoost, RSF, Enet, plsRcox, SuperPC, survival-SVM, GBM	AUC (1-yr - 0.67), (2-yr - 0.76), (3-yr - 0.72)
Proposed a 9-lncRNA-based framework for the prognosis of distant metastasis in LA-NPC with external validation/[48]	Nasopharyngeal carcinoma	Tumor tissue	560 samples (542 LA-NPC, 18 healthy controls)/Yes/Yes	Microarray profiling, RT-qPCR validation, univariate Cox and LASSO regression	LASSO Cox regression	AUC (0.78)
Developed 16 IRLS for prognosis and therapy guidance in colorectal cancer/[49]	Colorectal cancer	Tumor tissue	2509 samples from 17 public datasets & in-house cohort/Yes/No	Batch correction, WGCNA, ImmLnc pipeline, Lasso and Stepwise Cox	LASSO and Stepwise Cox	AUC (1-yr - 0.78), (3-yr - 0.76), (5-yr - 0.79)

In [45], a prognostic tool for gastric cancer was developed using lncRNAs associated with disulfidptosis (a type of cell death induced by disulfide stress). Based on the 371 gastric cancer patients sourced from the TCGA-STAD, 5 significant disulfidptosis-related lncRNAs (DRLs) were identified using co-expression analysis as well as LASSO Cox regression and served as the basis for constructing a risk score model that successfully stratified patients into high- and low-risk groups. The model proved to have independent prognostic performance with ROC AUCs of 0.720 (1-year), 0.646 (3-year), and 0.689 (5-year), and the validity was further checked using Kaplan–Meier curves, C-index evaluation, and nomogram calibration. The functional experiments involving qRT-PCR, Western blot analysis, as well as proliferation experiments served as affirmation of the key genes’ biological significance and identified potential therapy targets. The key contribution was the incorporation of a biologically rooted ML-based prognostic tool using disulfidptosis, which is a rarely used process in clinical models. However, the uniqueness of disulfidptosis as a mechanistic axis, though intriguing, remains underexplored beyond correlative GO/KEGG insights, necessitating prospective validation, deeper mechanistic interrogation, and assessment of performance in ethnically diverse populations before translational adoption in clinical settings.

Zhang *et al.* [46] constructed a disulfidptosis-associated gene (DPAG) prognostic model for gastric cancer using transcriptomic data from TCGA (403 samples) and GEO (433 samples) database. Consensus clustering of 16 DPAGs identified three molecular subtypes, with subtype C showing the poorest overall survival. LASSO-Cox and multivariate Cox regression across 1958 differentially expressed genes yielded 19 independent prognostic factors, from which a continuous DPAG score was derived. High DPAG scores independently predicted poor OS across training and validation sets, with time-dependent AUCs ranging from 0.66 to 0.73 and a C-index superior to standard clinicopathological variables. The DPAG score additionally correlated with immune infiltration, MSI status, TMB, and predicted differential drug sensitivity. Single-cell RNA sequencing confirmed DPAG signature expression across major cell subpopulations, with highest enrichment in epithelial cells and macrophages. In vitro knockdown of APC11, one of the 19 prognostic genes, suppressed proliferation and migration in two GC cell lines, supporting its oncogenic role. Limitations of this study include absence of prospective or independent wet-lab validation, and modest AUC values that constrain clinical utility relative to established staging systems.

The study [47] aimed to construct a robust ML-based model based on DRLs to stratify lung adenocarcinoma (LUAD) patients for survival prediction and therapeutic guidance using 916 patients’ transcriptomic and clinical data sourced from TCGA and GEO (GSE31210, GSE30219, GSE50081). From an initial pool of 708 DRLs, 27 prognostically significant lncRNAs were identified using univariate Cox regression analysis and incorporated into 10 ML models. The optimal model, i.e. Elastic Net (α = 0.2), achieved the highest performance across datasets (C-index >0.67; AUC up to 0.76). High- and low-risk groups exhibited distinct survival outcomes, immune profiles, mutational burdens, and treatment sensitivities. Notably, LINC01003 and LINC00857 emerged as key regulatory nodes through ceRNA and single-cell analysis. The study’s main contribution was ML-derived DRL signatures, supported by multi-omics validation and therapeutic relevance. Nonetheless, the modest predictive metrics, particularly a sub-0.7 C-index, reliance on retrospective, array-based GEO meta-cohorts, and the relatively high dimensionality of 27 lncRNAs may impede translational assay design.

The study [48] presented an immune-associated 9-lncRNA biomarker model for predicting distant metastasis in locoregionally advanced nasopharyngeal carcinoma (LA-NPC) patients. Derived through microarray screening and refined via RT-qPCR and LASSO regression in a 177-patient training cohort, the proposed model effectively stratified patients into high- and low-risk groups. Its predictive power was validated in two independent cohorts and outperformed traditional prognostic tools such as TNM staging and EBV DNA levels. Functionally, the lncRNAs were tightly linked to immune regulation and lymphocyte infiltration, suggesting a key role in tumor immune evasion. By integrating transcriptomic profiling, ML, and immune pathway analysis, the study delivers a clinically actionable and biologically insightful model for guiding personalized treatment in nasopharyngeal carcinoma.

The study [49] presented a significant advancement in colorectal cancer prognosis through a 16-immune-derived lncRNA signature (IRLS) coupled with a comprehensive ML framework. By integrating immune infiltration profiles with large-scale transcriptomic data from over 2500 patients across 17 public datasets and an in-house cohort, the authors established a prognostic tool with high generalizability. The use of diverse ML algorithms, including LASSO and stepwise Cox regression, along with leave-one-out cross-validation, demonstrates a strong methodological framework. IRLS consistently outperformed 109 published gene signatures in predicting overall and relapse-free survival and exhibited compelling predictive utility for chemotherapy and immune checkpoint inhibitor responses, particularly pembrolizumab. However, a major limitation was the study’s reliance on retrospective datasets without prospective clinical validation. Additionally, while the statistical and predictive strengths were clear, the biological functions of several lncRNAs in the signature remain unexplored, and the practicality of deploying a 16-lncRNA panel in clinical workflows was not addressed.

### circRNA, ML, and cancer

In this subsection, two most recent and impactful studies that used circRNA and ML in cancer diagnostics are critically evaluated (Table 5).

**Table 5:** Summary of cancer prognosis and diagnosis studies using circRNA and ML.

Study design/reference	Cancer type	Sample matrix	Sample size/dataset availability/code availability	Preprocessing adopted	Machine learning model	Performance metrics
Developed BEISA system and 4-circRNA-based model achieving strong performance in early and advanced GC detection/[50]	Gastric cancer	Plasma EVs	110 samples (Early GC, Advanced GC, healthy donors)/No/No	In situ analysis using DNA rectangular frameworks; ML-assisted multimodal signal analysis	LDA	AUC (0.93), sensitivity (0.87), specificity (0.90)
Designed a sensitive electrochemical platform for detecting sEV-circRNAs and proposed 4-circRNA-based model for stratifying GC stages/[51]	Gastric cancer	Plasma sEVs	90 samples (30 healthy, 32 Stage I-II GC, 28 Stage III-IV GC)/No/No	RNA extraction, RT-qPCR, signal amplification using DT-CHA with TDA tags, electrochemical sensing	MLP, SVM, RF, LDA	AUC (0.91), accuracy (0.96), sensitivity and specificity not reported

The study [50] aimed to develop a non-invasive, high-precision diagnostic tool for GC by combining bimodal detection of extracellular vesicle (EV)-associated circRNAs with ML analytics. Data were obtained from 110 participants across three cohorts, including healthy donors and GC patients at different clinical stages. EVs were isolated from plasma and characterized using established imaging and molecular techniques. The authors introduced the bimodal EV-circRNA *in situ* analyzer (BEISA), a dual-mode analyzer capable of providing dual outputs in fluorescence and electrochemical modes via a DNA framework-guided hybridization chain reaction. Four circRNAs (circ-URI1, circ-YME1L1, circ-CWF19L1, circ-OGA) were selected as screening biomarkers. ML using linear discriminant analysis (LDA) classified samples with 88.0% accuracy, 90.0% specificity, and an AUC of 0.925. The non-invasive, amplification-enhanced design obviated EV lysis and RNA extraction, and the bimodal readouts provided internal cross-validation. However, the small sample size (*n* = 110) and reliance on small, single-center cohorts may limit generalizability. While the framework proposed in the study is methodologically sound, comparisons to existing clinical standards (e.g. PET/CT, gastroscopy) were lacking, and potential preanalytical variability in EV isolation protocols remained unaddressed. The technical complexity and requirement for specialized instrumentation may hinder near-term clinical adoption.

The authors of [51] presented a dual-signal amplification electrochemical biosensor (combining TDA-tagged AuNPs and DT-CHA) to detect four sEV-circRNAs with attomolar sensitivity (LOD 153.1 aM) and a broad linear range (1 fM-1 nM). An MLP classifier attained excellent discrimination of healthy versus early-stage GC (100% accuracy during training) with 96% accuracy in an independent cohort. Their integration of nanostructured DNA frameworks and ML demonstrated cutting-edge assay design and yielded strong concordance with qRT-PCR. However, the study’s small sample size (*n* = 90) and single-center recruitment raise substantial concerns about cohort heterogeneity and overfitting. The complexity of ultracentrifugation-based sEV isolation, specialized instrumentation, and multilayered signal amplification impedes routine clinical adoption, and the reliance on a four-marker panel (without head-to-head comparison to existing modalities such as endoscopic biopsy or plasma protein markers) may limit assessment of added value. Prospective, multicenter validation streamlined assay workflows, and cost–benefit analyses will be essential to substantiate translational viability in the real-world settings.

### piRNA, ML, and cancer

piRNA-based cancer detection using ML is not prevalent in the literature. However, piRNA represents a potential ncRNA subclass which could be utilized in the diagnosis of cancers. In this subsection, two studies that used piRNA and ML in the detection of cancer are evaluated (Table 6).

**Table 6:** Summary of cancer diagnosis study using piRNA and ML.

Study design/reference	Cancer type	Sample matrix	Sample size/dataset availability/code availability	Preprocessing adopted	Machine learning model	Performance metrics
Developed a 2-piRNA-based diagnostic model for LUAD integrated with LDCT nodule classification in a multicenter cohort/[52]	Lung adenocarcinoma	Serum	1521 samples (1033 LUAD, 89 benign nodules, 399 healthy controls) from 3 centers/No/No	*z*-score normalization, DE analysis, fold-change and FDR filtering, SMOTE for class balancing	RF, LR, SVM, GB, KNN, Naïve Bayes, ANN, Elastic Net, Decision Tree	AUC (0.87), Sensitivity (0.91), Specificity (0.71)
Proposed piRNA descriptor-based ML model for colorectal cancer diagnosis with good generalization to independent data/[53]	Colorectal cancer	Serum	13 differentially expressed piRNAs; external validation with 7 CRC-related piRNAs and 9 breast cancer related piRNAs/Partial/No	Descriptor creation (1020), Descriptor reduction (27)	Naïve Bayes Multinomial, MLP, RF, AdaBoostM1, Decision Table	AUC (0.99), sensitivity (0.96), specificity (0.97)

Liang *et al*. [52] conducted a multicenter study applying piRNA-based ML for non-invasive lung adenocarcinoma (LUAD) detection. Using 1521 serum samples from 1473 participants across three institutions, they identified two circulating piRNAs (piR-hsa-8393202 and piR-hsa-8429916) as diagnostic biomarkers through paired tissue-serum omics profiling and qPCR validation. Among nine ML algorithms benchmarked, random forest achieved internal validation AUC of 0.87 (sensitivity 82.2%, specificity 82.4%), and external validation AUC of 0.87 (sensitivity 90.8%, specificity 71.4%). Furthermore, a pulmonary nodule classifier combining both piRNAs with carcinoembryonic antigen (CEA) substantially outperformed CEA alone, and functional knockdown experiments in two cell lines supported the pro-oncogenic roles of both biomarkers. The key strengths of their study include the multicenter design, rigorous preprocessing, and adherence to TRIPOD standards. However, the limitations of this study include a retrospective design with selection bias toward treatment-naive surgical candidates, a modest NPV constraining rule-out utility, and incompletely characterized molecular mechanisms.

The authors of [53] aimed to evaluate piRNAs as non-invasive biomarkers for colorectal cancer (CRC) using an ML-based diagnostic. Serum-derived datasets containing 13 CRC-associated piRNAs were used; from which 1020 numerical sequence descriptors were extracted, including nucleotide composition, motif patterns, and structural features. Feature selection using InfoGain reduced the descriptors from 1029 to 27, and ML classifiers were trained using WEKA. The Naïve Bayes multinomial model achieved 96.4% accuracy on training data and 85.7% on external CRC validation, but only 44.4% on unrelated piRNAs, indicating strong specificity. The study’s key contribution was demonstrating that piRNA sequence patterns could enable fairly high-accuracy CRC detection through ML. Nonetheless, the narrow piRNA subset and lack of in vivo validation underscore the need for larger, clinically validated datasets.

### Comparative landscape among miRNA, lncRNA, circRNA, and piRNA-based cancer diagnosis and prognosis

Despite rapid progress in ncRNA-ML-based cancer diagnosis and prognosis, the four major subclasses of ncRNA, i.e. miRNA, lncRNA, circRNA, and piRNA differ significantly in clinical readiness, diagnostic performance, and ML-related challenges.

miRNAs remain the most clinically advanced and extensively validated subclass, and among all four subclasses, they are the closest to routine clinical translation. Their abundance in serum, plasma, and other biofluids, moderately stable expression profiles, well-established quantification platforms such as qPCR, microarrays, and next-generation sequencing, and the availability of large, well-annotated public datasets (GEO, TCGA, etc.) are the most important enablers. Consequently, miRNA-based ML models consistently demonstrated high diagnostic performance across multiple cancer types, often supported by standardized preprocessing pipelines and external validation cohorts. However, even in this relatively mature domain, challenges such as cohort bias, retrospective study design, and limited mechanistic interpretability persist and must be resolved before full clinical deployment.

In contrast, lncRNA-based approaches offer strong biological specificity in cancer prognostic applications. However, a critical practical limitation is that all lncRNA studies reviewed here relied exclusively on tumor tissue obtained through invasive surgical or biopsy procedures, fundamentally constraining their applicability to post-surgical settings and precluding their use in early-stage non-invasive screening. Beyond this accessibility barrier, the very low expression levels and high tissue specificity of lncRNAs create a high-dimensional and often noisy feature space. The prognostic performance of existing lncRNA models, with AUC typically in the range of 0.6 to 0.8, also requires further improvement before these models can be considered clinically competitive. Expanded biomarker discovery efforts across diverse cancer types and the development of less invasive lncRNA quantification strategies will therefore be a prerequisite for meaningful clinical translation.

circRNAs occupy a particularly intriguing position owing to their exceptional structural stability conferred by their covalently closed loop architecture, which renders them highly resistant to exonucleolytic degradation in biofluids and makes them attractive candidates for non-invasive ML-based cancer diagnostics. However, most published investigations relied on small, single-center cohorts and technically demanding detection instrumentation, currently limiting reproducibility, scalability, and cross-study comparability. Critically, publicly available circRNA expression datasets for cancer remain sparse, and systematic biomarker discovery studies spanning diverse cancer types are still lacking. Expanding the circRNA public data ecosystem and conducting large-scale multicenter discovery studies are therefore essential prerequisites before robust and generalizable ML models can be developed.

Finally, due to the scarcity of well-annotated somatic expression datasets and an incomplete understanding of their functional roles outside germline tissue, piRNA-based cancer diagnosis and prognosis studies are not that prevalent. The near-total absence of large-scale, publicly available cancer-specific piRNA expression resources represents the single greatest barrier to ML model development in this subclass, and addressing this data deficit must be recognized as the field’s foremost upstream priority before any sophisticated modelling strategy can be meaningfully pursued.

Overall, miRNAs currently lead the landscape and are approaching clinical translatability [35], while circRNAs offer compelling innovation potential driven by their unmatched biofluid stability. lncRNAs remain vital for tissue-based prognostic applications but require improved diagnostic performance, less invasive detection strategies, and broader cancer-type coverage. piRNAs hold considerable potential for future discovery but demand urgent investment in somatic dataset generation.

## ncRNA-ML-based cancer diagnostics: roadmap, technical hurdles and emerging opportunities

Despite substantial advances in ncRNA-based ML models for cancer diagnosis and prognosis, their translation into routine clinical practice remains limited. Bridging this gap requires a structured framework spanning data generation, model development, validation, and clinical deployment.

The existing experimental paradigm in ncRNA investigations typically involves profiling a multitude of transcripts across a limited number of patient specimens, yielding datasets that are both small in sample size and vast in feature dimensionality, a common phenomenon referred to as the ‘curse of dimensionality’. Such high-dimensional data increase the risk of overfitting, where models are very likely to capture noise in the training cohort which eventually undermine the models applicability to more diverse patient populations. Although no universal sample-size threshold exists, and the optimal requirement will inevitably vary depending on the number of features, cancer type, and study design, we suggest that diagnostic models should be trained on at least 300 to 500 samples per class as a pragmatic working benchmark, encompassing diverse demographic and clinical characteristics, with control groups including clinically relevant comparators such as benign conditions rather than healthy individuals alone to minimize spectrum bias. Longitudinal sampling should be incorporated wherever possible to capture temporal variability in ncRNA expression and cancer progression.

ML algorithms, like most analytical approaches, depend on high quality, dependable datasets. In ncRNA studies, a substantial fraction of data may be missing due to technical failures, while genuinely non-expressed transcripts yield undetectable values that require special treatment [54]. Such gaps and zeros can undermine model stability and predictive accuracy if left unaddressed. Therefore, rigorous preprocessing including sophisticated imputation strategies is essential. Following imputation, quantile normalization is essential to render expression values comparable across samples [55], and batch effects arising from multi-center or multi-platform data collection must be corrected using tools such as ComBat prior to any ML modelling [56]. Furthermore, as skewed class proportions within ncRNA-based datasets can bias classifiers toward majority classes, techniques such as resampling, synthetic minority oversampling (SMOTE), or cost -sensitive learning can help rebalance the training data [57, 58]. However, the systematic development of curated, cancer-specific expression profiles spanning diverse cancer types, and patient demographics should be prioritized, especially for piRNA, as no ML-based strategy can compensate for the absence of biologically reliable and clinically representative training data.

Equally critical is the careful management of feature dimensionality. Cluster-based reduction strategies can constrain the combinatorial explosion of candidate panels preserving predictive accuracy while reducing computational burden [59]. Feature selection methods based on ensemble algorithms such as RF, XGBoost, often preceded by a filtering step to discard highly correlated ncRNAs, have likewise proven effective at identifying discriminative biomarkers in high-dimensional datasets [60, 61]. From an implementation perspective, streamlined biomarker panels not exceeding 10 ncRNAs are preferable, as they will reduce assay complexity, turnaround time, and cost significantly. Feature selection strategies should therefore balance predictive accuracy with biological interpretability and technical reproducibility. In addition, feature stability analyses via bootstrapping and stability selection must be routinely employed to ensure selected biomarkers remain robust across resampled datasets.

An overly complex ncRNA-based classifier, weighed down with an abundance of features, often proves of limited practical value, since its outputs become biologically opaque. Building clinician trust in AI-driven ncRNA diagnostics requires moving beyond post-hoc feature importance scores to developing interpretability frameworks embedded within the clinical workflow itself. SHAP (SHapley Additive exPlanations) values [62] can be rendered as patient-level waterfall plots showing each ncRNA’s contribution to an individual’s risk score, enabling oncologists to interrogate whether the model’s decision aligns with known biological mechanisms. Local Interpretable Model-Agnostic Explanations (LIME) [63] offers an alternative for explaining individual predictions in a clinician-interpretable linear approximation. At the system integration level, clinical decision support tools embedding these models should present uncertainty estimates such as calibrated probability outputs with 95% confidence intervals alongside predictions, flag cases where the model is operating outside its training distribution, and recommend referral to specialist review in ambiguous cases.

The comparative evaluation of ML approaches in ncRNA-based cancer prognosis and diagnosis remains scarce, and the choice of an optimal model is typically driven by empirical trial rather than a priori rationale [64]. Systematic benchmarking on identical ncRNA cohorts would clarify which algorithms are best suited to specific research aims [65]. In this context, ensemble frameworks that unify several models into a single predictor should be employed in ncRNA-based cancer detection as an essential tactic for enhancing robustness and predictive power [66, 67].

High-throughput multi-omics platforms now make it feasible to integrate ncRNA expression profiles with genomic, proteomic, and metabolomic layers. Such integration via ML frameworks can yield a more holistic depiction of the molecular landscape underpinning cancer onset and progression [68], and multimodal models are better positioned to capture biological complexity and improve predictive performance across heterogeneous patient populations [69].

Deep neural architectures, with their hierarchical feature-extraction capabilities, excel at uncovering complex, nonlinear associations between ncRNA profiles and biological phenotypes. They have been successfully applied to ncRNA identification, interaction prediction, mechanistic interpretation, and disease-association [70], although their application to screening cancers remains nascent. Meanwhile, reinforcement learning, though still nascent within the ncRNA domain, offers a framework for iteratively optimizing biomarker selection and forecasting novel ncRNA disease linkages through reward-driven exploration [71, 72]. A systematic appraisal of these advanced methodologies may yield critical insights and accelerate breakthroughs.

The landscape of ncRNA-based cancer detection must also cope with broader, non-AI/ML specific challenges spanning methodological, technical, and experimental domains. Rigorous validation is indispensable for establishing clinical utility. At a minimum, studies should incorporate internal validation through cross-validation or bootstrapping, followed by independent external validation using geographically or institutionally distinct cohorts. Ultimately, prospective validation in real-world clinical settings is essential to assess true clinical performance. Notably, the absence of prospective validation remains one of the most significant shortcomings of the current literature. Artificial case–control studies, though valuable for explaining molecular pathways, frequently inflate performance estimates, which could be misleading. Hence, validation in authentic clinical cohorts comprising patients with suspected pathology is of utmost importance. Without it, reported AUC values substantially overestimate true real-world diagnostic performance. Finally, the selection of readily obtainable and routinely processed biospecimens such as urine or saliva warrants greater emphasis, particularly when targeting special populations such as pediatric patients, to ensure practical and non-invasive implementation.

Economic and operational feasibility are critical yet constantly underreported determinants of clinical adoption. Even highly accurate ncRNA-based ML models will not achieve real-world implementation if they are prohibitively expensive or technically impractical for routine laboratory use. A few studies reviewed in this article achieved impressive diagnostic performance using relatively large ncRNA panels (100-miRNA panel [38], 79-miRNA panel [40], 39-miRNA panel [41]). However, panel size of this magnitude poses substantial barriers to clinical translation. Quantifying such a high number of candidate ncRNAs demands multiplexed assay design, extensive primer optimization, and significant per-run reagent costs that are very unlikely to be sustainable for population-level screening. The total cost per test, encompassing reagents, instrumentation, computational infrastructure, and labor, must therefore be systematically evaluated alongside conventional diagnostic performance metrics, and should be optimized accordingly to ensure a balance between diagnostic performance and cost-effectiveness.

Embracing open science principles is vital to foster knowledge advancement and generate broadly applicable datasets. The public dissemination of raw data, analytical code, and experimental protocols promotes transparency and reproducibility in ncRNA-ML investigations. Replication capacity is the foundation of scientific validity, and shared datasets empower researchers to integrate different data modalities, strengthen statistical power, and facilitate the reinterpretation of existing resources. Open data practices further catalyze collaboration and accelerate discovery.

A suite of dedicated, publicly accessible repositories has been established to standardize and curate ncRNA research outputs. For instance, RNAcentral (https://rnacentral.org) [73] provides a comprehensive catalog of published ncRNA sequences. Complementing this resource, miRBase [23], NONCODE [29], circRNADb [30], and piRBase [31] provide specialized repositories for miRNAs, lncRNAs, circRNAs, and piRNAs, respectively. RNALocate [74] facilitates systematic exploration of ncRNA subcellular localization, supporting mechanistic investigations into spatially regulated RNA functions.

Broad-spectrum data portals such as GEO (https://www.ncbi.nlm.nih.gov/geo), TCGA (https://www.cancer.gov/ccg/research/genome-sequencing/tcga), and the EMBL EBI (https://www.ebi.ac.uk/) provide extensive access to curated datasets. Platforms such as protocols.io (https://www.protocols.io) support the secure dissemination, version control, and standardization of experimental protocols. In addition to this, code-sharing infrastructures, most notably GitHub (https://github.com), facilitate the open exchange of analytical workflows and software tools. Community platforms such as Kaggle (https://www.kaggle.com) further foster an open ecosystem for the dissemination of ML models, datasets, and computational code while connecting researchers through organized competitions and collaborative challenges. By evaluating algorithms across heterogeneous datasets and problem domains, these initiatives accelerate model generalizability, improve robustness, and facilitate the identification of discriminative features.

Beyond the four ncRNA subclasses, i.e. miRNA, lncRNA, circRNA, and piRNA, critically evaluated in this review, mounting evidence supports the cancer diagnostic and prognostic potential of additional ncRNA subclasses such as snoRNAs [75], tRNA-derived RNA fragments (tRFs) [76], YRNAs [24], and orphan non-coding RNAs (oncRNAs) [77], each of which merits serious consideration as an emerging frontier for ncRNA-ML-based cancer diagnostics. Notably, Karimzadeh *et al.* [78] developed Orion, a multi-task deep generative AI model based on variational autoencoders, to analyze circulating oncRNAs from serum samples of 1050 non-small cell lung cancer (NSCLC) patients and matched controls. This groundbreaking study achieved 94% sensitivity at 87% specificity across all cancer stages and outperformed all conventional methods on held-out validation datasets by over 30% in sensitivity, demonstrating oncRNA capability in cancer diagnosis through the robust and generalizable ML model Orion.

## Conclusion

Cancer diagnosis and prognosis remain paramount for reducing disease burden and improving patient survival outcomes. Yet, early diagnosis continues to be hindered by tumors remaining asymptomatic during initial stages, comorbidities obscuring disease presentation, and existing screening strategies lacking sufficient sensitivity and specificity. In this context, ncRNAs have emerged as highly promising molecular biomarkers owing to their extensive regulatory roles in tumor biology, detectable dysregulation across diverse malignancies, and stability in biofluids. Several studies report highly sensitive and specific ncRNA signatures capable of detecting challenging malignancies such as NSCLC and hepatocellular carcinoma, while also distinguishing pancreatic cancer from pancreatitis with ‘high diagnostic’ fidelity [42, 44, 78–80].

This review critically evaluated ML-based cancer prognosis and diagnosis studies involving miRNAs, lncRNAs, circRNAs, and piRNAs. miRNAs represent the most mature and clinically advanced subclass, while circRNAs demonstrate substantial promise owing to their exceptional biofluid stability. lncRNA-based frameworks show meaningful prognostic value primarily in tissue-based applications, and piRNAs remain underexplored due to well-annotated somatic datasets. Technological advances have further ‘strengthened’ ncRNA detection and analysis. Platforms like NanoString’s nCounter®, microRNA panel, and next-generation RNA sequencing now ‘provide improved sensitivity, specificity, and multiplexing capability compared with conventional RT-qPCR.’ Despite this progress, clinical translation remains impeded by biological heterogeneity, retrospective study designs, inadequate prospective and multicenter validation, and the biological opacity of many current ML frameworks.

Future progress in ncRNA-ML-based cancer diagnosis and prognosis will depend on the generation of large, diverse, clinically representative datasets supported by rigorous prospective validation frameworks. Greater emphasis must also be given on interpretable ML, multimodal data integration, feature stability analysis, and the development of streamlined biomarker panels that are economically feasible for clinical implementation. Alongside, interdisciplinary collaboration across molecular biology, bioinformatics, artificial intelligence, and clinical oncology, reinforced by ‘open-source data sharing, and adoption of standardized experimental and computational protocols will further facilitate realizing’ the full potential of ncRNA-based ML diagnostics in precision oncology.

**Key Points**

- ncRNAs are stable, biofluid-accessible regulators of gene expression that offer ML-driven cancer diagnostics non-invasively.
- To our knowledge, this is the first review to critically evaluate ML-driven cancer diagnosis and prognosis studies across four major ncRNA subclasses: miRNA, lncRNA, circRNA, and piRNA.
- miRNAs lead clinical translatability, supported by large, annotated datasets and relatively standardized workflows; lncRNA and circRNA approaches are rapidly emerging, while piRNA-based detection remains nascent due to limited annotated datasets.
- Despite high diagnostic performance in retrospective studies, major clinical translational barriers exist, including smaller cohorts, lack of prospective multicenter validation, and biologically opaque ML models.
- Future ncRNA-ML frameworks must adopt explainable AI, streamlined ncRNA panels, harmonized preprocessing standards, and multimodal integration with clinical variables to achieve routine clinical deployment.

## Data Availability

No new data were generated or analyzed in this review. All data referred to are from published literature and are cited appropriately in the reference list.
